# Bowel Obstruction Secondary to Spontaneous Knot Formation of Ventriculoperitoneal Shunt

**DOI:** 10.7759/cureus.31236

**Published:** 2022-11-08

**Authors:** Meera R Laxman, Christopher A Gegg, Tamarah Westmoreland

**Affiliations:** 1 Pediatric Surgery, Nemours Children’s Hospital, Orlando, USA; 2 Neurosurgery, Nemours Children’s Hospital, Orlando, USA; 3 Pediatric Surgery, University of Central Florida College of Medicine, Orlando, USA

**Keywords:** bowel obstruction, general surgery, pediatric surgery, ventriculoperitoneal shunt, sponteanous knot formation

## Abstract

Ventriculoperitoneal (VP) shunts are frequently placed for the treatment of hydrocephalus. Shunt complications are a common occurrence typically involving infection, disconnections, or blockages. Abdominal complications involving the intraperitoneal portion of the catheter are rare. Spontaneous peritoneal knot formation involving the bowel with subsequent obstruction is even rarer. Spontaneous knot formation of a VP shunt is also not commonly seen in the adult population. In this report, we present the case of an 18-year-old male with cerebral palsy and hydrocephalus requiring VP shunt placement who developed a spontaneous knot leading to bowel obstruction requiring emergency laparoscopic surgery.

## Introduction

Ventriculoperitoneal (VP) shunt placement is crucial for the management of hydrocephalus in the pediatric and adult populations [1,2]. Although it is a common neurosurgical procedure, there are numerous complications associated with shunt placements. About 40% of shunts fail within the first year of placement for a variety of reasons [3]. These include infection, blockages (either proximally or distally) due to shunt placement/migration or adhesions, formation of pseudocysts, and valve malfunction. The most common complications are infection and shunt malfunction from an obstruction [3,4]. The distal portion of shunts can also migrate into the heart, scrotum, and bowel and can cause bowel perforation as well as distal catheter entrapment [1,4,5]. Despite the routine use of VP shunts, the most common complications involving the abdomen include ascites due to cerebrospinal fluid (CSF) leaks, hydrocele formation, infection, shunt migration and extrusion, and formation of loculated cysts [2]. Spontaneous knot formation of the catheter is extremely rare, and subsequent bowel obstruction and/or necrosis is even rarer [4,6,7]. With only three additional cases reported in the pediatric population, most of these cases occurred in patients with a history of repetitive manipulation of the catheter [3,6,8]. This case report adds to the knowledge of the VP catheter knot formation and its laparoscopic treatment.

## Case presentation

An 18-year-old male with a complex medical history including cerebral palsy, hydrocephalus (requiring VP shunt placement shortly after birth), global developmental delay, seizure disorder, and hearing loss presented to the emergency room with chief complaints of headaches, nausea, and difficulty with balance. Prior to the presentation to the emergency department (ED), he had undergone surgery for shunt valve replacement due to similar symptoms a few days before this admission. Prior to his most recent shunt revision, he previously had two replacements as a toddler, one of which was a distal revision.

During his ED visit, the patient’s mother reported that a few days earlier, she noticed swelling on the right lateral neck, near the VP shunt tract, which had increased in size with warmth and tenderness. In the ED, shunt series X-rays were normal, shunt series computed tomography (CT) was normal, and an abdominal ultrasound (US) for evaluation of the distal shunt did not show signs of pseudocysts. Of note, he did have a history of constipation that had recently worsened despite daily use of Miralax and was also complaining of new-onset abdominal pain.

Due to continued symptoms despite shunt revision two weeks prior, the patient was admitted for the management of his headaches. On hospital day two, the patient was noted to have purulent drainage from his scalp incision with fluctuance. Due to also being febrile, he was preemptively started on vancomycin and ceftriaxone.

Subsequently, he was taken to the operating room (OR) for externalization of his VP shunt by neurosurgery. His prior incision was opened, and purulence was noted, likely the cause of his fever. After drainage of the purulence, the area was thoroughly irrigated. The ventricular catheter was disconnected from the proximal valve, and a brisk flow of CSF through the ventricular catheter was noted. Next, the removal of the peritoneal catheter was attempted blindly. Initially, the peritoneal catheter moved freely when pulled; however, the removal was then met with significant resistance. The decision was made to cut the catheter and remove the ventricular portion with plans to retrieve the abdominal portion subsequently in the same operation. After successful removal and externalization of the ventricular portion, attention was turned to the previous abdominal incision which was excised to the peritoneal catheter. The catheter was easily identified. The proximal catheter was completely withdrawn, but the intraperitoneal portion remained fixed and was unable to be freed. The general surgeon was then consulted during that same procedure for retrieval of the remainder of the catheter.

**Figure 1:**
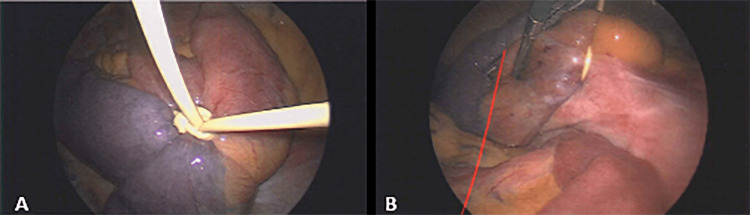
(A, B) Intraoperative imaging showing ventriculoperitoneal shunt catheter knot cinched around the bowel causing ischemia to the bowel from different angles.

**Figure 2:**
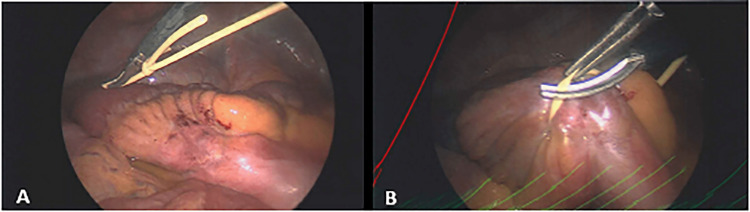
(A, B) Intraoperative imaging showing the cutting of the knotted ventriculoperitoneal shunt catheter.

**Figure 3:**
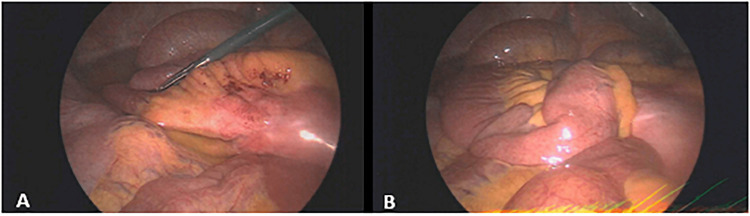
(A, B) Intraoperative imaging showing the return of blood supply to the affected bowel.

Upon entry into the abdomen laparoscopically, the intraperitoneal portion of the VP shunt was identified and observed to be wrapped around a segment of the small bowel. A knot in the catheter was observed to be constricting blood flow (Figure 1A). It appeared to be a slip-knot that was unknowingly tightened further during attempted removal. The knot and further cinching of it resulted in ischemia to a portion of the small bowel (Figures 1A, 1B). The intraperitoneal shunt was removed with laparoscopic scissors (Figures 2A, 2B), and the blood supply to the small bowel was released allowing an immediate return of intestinal color and viability (Figures 3A, 3B).

The patient was transferred to the pediatric intensive care unit for postoperative management and made a full recovery. Postoperatively, he remained afebrile and stable without emesis. He was tolerating a clear liquid diet on postoperative day one.

## Discussion

Spontaneous knot formation of a VP shunt is an extremely rare complication. Subsequent bowel obstruction with or without necrosis is an even rarer complication. However, it should be considered when experiencing difficult catheter removal met with resistance or with a history of frequent shunt manipulation [7,9]. While some neurosurgeons opt to cut the VP shunt at the skin level and allow the abdominal portion of the catheter to remain if experiencing difficulties during removal, there have been other cases of the peritoneal portion of the shunt involving the bowel [7,8]. Furthermore, while knot formation is thought to be spontaneous, some research suggests that it could originate from repetitive tugging causing a loop of the catheter to cinch around the bowel, restrict blood flow, and ultimately lead to bowel ischemia [7].

Excessive catheter length is thought to be a contributing factor to knot formation [3,8,10]. Especially in children, depending on the age of the initial catheter placement, extra length is added to account for patient growth. A smaller catheter diameter with a highly elastic material composition is also hypothesized to contribute to knot formation [8]. Additionally, patient factors can also play a role. These include intra-abdominal adhesions from previous surgical procedures and increased bowel peristalsis [8].

A physician should always have a high index of suspicion for knot formation in the peritoneal portion of a VP shunt with involvement of the bowel if a patient experiences signs of obstruction. These signs include vomiting, abdominal pain/discomfort, abdominal distention, and radiography with suspicion of obstruction following difficult shunt removal or resistance during manipulation [11]. This case report presents one of only four cases in the older pediatric/adult population at the time of the literature review [3,6,8].

## Conclusions

Spontaneous knot formation in the peritoneal portion of a VP shunt is a rare complication. It is an extremely important consideration when experiencing difficult shunt removal or performing frequent manipulation of the shunt. Radiography is highly recommended immediately after manipulation to assess for any abdominal complications. Clinicians should be on high alert for signs and symptoms of bowel obstruction/ischemia due to potential knot formation if the catheter feels tethered and is not easily removed. In such cases, in addition to supportive imaging, it is prudent to perform an exploratory laparoscopy or laparotomy to assess bowel compromise with regard to spontaneous knot formation in a VP shunt.
